# Goal-Concordant Care After Severe Acute Brain Injury

**DOI:** 10.3389/fneur.2021.710783

**Published:** 2021-09-17

**Authors:** Rachel Rutz Voumard, Kaley M. Dugger, Whitney A. Kiker, Jason Barber, Gian Domenico Borasio, J. Randall Curtis, Ralf J. Jox, Claire J. Creutzfeldt

**Affiliations:** ^1^Department of Neurology, Harborview Medical Center, University of Washington, Seattle, WA, United States; ^2^Department of Palliative and Supportive Care, Lausanne University Hospital, University of Lausanne, Lausanne, Switzerland; ^3^Division of Pulmonary, Critical Care and Sleep Medicine, University of Washington, Seattle, WA, United States; ^4^Cambia Palliative Care Center of Excellence, University of Washington, Seattle, WA, United States; ^5^Department of Neurosurgery, Harborview Medical Center, University of Washington, Seattle, WA, United States; ^6^Institute of Humanities in Medicine, Lausanne University Hospital, University of Lausanne, Lausanne, Switzerland

**Keywords:** neuropalliative care, severe acute brain injury, goal-concordant care, shared decision-making, palliative care

## Abstract

**Background:** Patients with severe acute brain injury (SABI) lack decision-making capacity, calling on families and clinicians to make goal-concordant decisions, aligning treatment with patient's presumed goals-of-care. Using the family perspective, this study aimed to (1) compare patient's goals-of-care with the care they were receiving in the acute setting, (2) identify patient and family characteristics associated with goal-concordant care, and (3) assess goals-of-care 6 months after SABI.

**Methods:** Our cohort included patients with SABI in our Neuro-ICU and a Glasgow Coma Scale Score <12 after day 2. Socio-demographic and clinical characteristics were collected through surveys and chart review. At enrollment and again at 6 months, each family was asked if the patient would prefer medical care focused on extending life vs. care focused on comfort and quality of life, and what care the patient is currently receiving. We used multivariate regression to examine the characteristics associated with (a) prioritized goals (comfort/extending life/unsure) and (b) goal concordance.

**Results:** Among 214 patients, families reported patients' goals-of-care to be extending life in 118 cases (55%), comfort in 71 (33%), and unsure for 25 (12%), while care received focused on extending life in 165 cases (77%), on comfort in 23 (11%) and families were unsure in 16 (7%). In a nominal regression model, prioritizing comfort over extending life was significantly associated with being non-Hispanic White and having worse clinical severity. Most patients who prioritized extending life were receiving family-reported goal-concordant care (88%, 104/118), while most of those who prioritized comfort were receiving goal-discordant care (73%, 52/71). The only independent association for goal concordance was having a presumed goal of extending life at enrollment (OR 23.62, 95% CI 10.19–54.77). Among survivors at 6 months, 1 in 4 family members were unsure about the patient's goals-of-care.

**Conclusion:** A substantial proportion of patients are receiving unwanted aggressive care in the acute setting after SABI. In the first days, such aggressive care might be justified by prognostic uncertainty. The high rate of families unsure of patient's goals-of-care at 6 months suggests an important need for periodic re-evaluation of prognosis and goals-of-care in the post-acute setting.

## Background

To provide goal-concordant care means to provide medical care that honors a patient's individual goals and values, and to align medical treatments with those goals-of care (1, 2). Recent studies suggest that prior documentation of preferences to limit life-sustaining treatment may reduce the likelihood of being admitted to an ICU in the last 6 months of life (3). Studies of the past decade have also shown that such values and goals can be difficult to assess prior to an illness and to translate into relevant goals-of-care (4, 5).

When patients are admitted to the hospital with severe acute brain injury (SABI), which includes stroke, traumatic brain injury and hypoxic-ischemic encephalopathy after cardiac arrest, they typically lack decisional capacity and rarely have had their goals-of-care previously documented. Consequently, their family members or other surrogate decision-makers are tasked to work with clinicians to make treatment decisions based on the patients' presumed goals (6). Treatment decisions in the acute setting of SABI often concern high-stakes decisions around whether to focus medical care on survival, including the use of life-sustaining treatment (LST) such as mechanical ventilation, artificial nutrition or hydration, or to focus on comfort, which may mean limiting LST and allowing the patient to die a more natural death (7, 8). Given the substantial uncertainty regarding both the patients' prognosis and their presumed goals-of-care, LST is often administered as a time-limited trial in order to gain a better understanding of the patient's trajectory, prognosis, and likely goals over a defined period of time (9, 10). Consequently, at the end of a period of a time-limited trial, the continued use of LST has to be re-evaluated (11). Prognostic uncertainty typically persists for months after SABI and can challenge ongoing decisions in the acute care and post-acute care setting (12).

Using a cohort of patients with SABI, the objective of this study was therefore to assess (1) patients' presumed goals-of-care as assessed by family members in the acute setting; (2) the frequency of family-assessed goal-concordant care and the patient and family characteristics associated with family-assessed goal-concordant care; and (3) whether and how these goals have changed 6 months later.

## Methods

### Study Design and Participants

The SuPPOrTT^*^ study is a prospective, observational, single-center cohort study that aims to better understand the needs of patients and family members after SABI. Patient participants were aged 18 years and older and hospitalized in the Neuro-ICU for SABI. We defined SABI as stroke (ischemic stroke, intraparenchymal hemorrhage, subarachnoid hemorrhage), hypoxic-ischemic encephalopathy after cardiac arrest (HIE), or traumatic brain injury (TBI). Our definition also included a Glasgow Coma Scale of 12 or less at enrollment after day 2. Eligible family participants were aged 18 years and older and spoke English adequately to complete surveys. For patients to be eligible, family members must have been available in person or by phone. Family member participants were primarily the surrogate-decision maker or, with the surrogate-decision maker's permission, the next close family member or friend, including spouse/partner, adult child, parent, sibling, or other close relative. After agreement of the clinical team in charge of the patient, family members were approached in person at the bedside or by phone and invited to participate in the study. The protocol was approved by the ethical review board of the University of Washington (STUDY 00003393).

### Outcomes

We were interested in three outcomes that were assessed through family surveys: (a) family assessment of patients' prioritized healthcare goals at enrollment; (b) the family-perceived priorities of the actual care that the patient was receiving at the time of enrollment; and (c) family assessment of patients' prioritized healthcare goals 6 months after SABI. We asked one family member per patient to state the patient's goals-of-care by using the following question that was adapted from the landmark Support study (13) (question a): “If your loved one were able and had to make a choice today, would he/she prefer a plan of medical care that focuses on extending life as much as possible, or would he/she want a plan of medical care that focuses on comfort, and would limit life-saving treatments?” Three response options were offered: (1) efforts to extend life as much as possible, (2) limit life-saving treatment and focus on comfort, or (3) unsure what they would choose. We then asked the family about the type of care they felt their family member was currently receiving, using the same three response options (question b). When care received was consistent with family-assessed goals-of-care (a = b), we considered the care to be goal concordant. Six months after enrollment, the first question was repeated verbatim in the follow-up survey that we sent to families of survivors by mail or email.

### Patient and Family Characteristics

To evaluate factors associated with each family-assessed prioritized healthcare goal as well as predictors of goal concordance at enrollment, we retrieved patient clinical and socio-demographic data retrospectively through the electronic health records (EHR), and families filled out a sociodemographic questionnaire regarding personal data. Patient characteristics included age; gender; race/ethnicity; disease category; clinical severity described with the APACHE score, and neurological severity described with Glasgow Coma Scale score (GCS). Family self-reported characteristics included age; gender; race/ethnicity; relationship to patient; level of education.

### Analysis

Data were collected using Research electronic data capture (REDCap) (14). Differences between demographic and other patient and family characteristics by prioritized healthcare goals were assessed for statistical significance using Kruskal-Wallis tests for continuous and ordinal variables and Fisher's exact tests for nominal variables. We used multivariate regression to examine how patient and family characteristics were associated with (1) each prioritized end-of-life goal (extending life, comfort, unsure; using nominal regression) and (2) goal concordance (using logistic regression). Multivariate models were constructed by starting with age and race as covariates regardless of significance, then putting in additional covariates one at a time using a forward selection algorithm until no remaining covariates could provide sufficient improvement, setting a threshold of p <.05 to enter. All statistical testing was two-sided, with no *post-hoc* adjustments for multiple comparisons given the exploratory nature of this study.

Alluvial diagrams were used to visualize the relationship between prioritized goals vs. care received, and between prioritized goals at enrollment vs. at 6 months, and constructed using RAWgraphs (15).

## Results

Of the 222 patients enrolled in our SuPPOrTT study, families answered the goal concordance questions for 214 in the acute setting at a mean of 5.1 (SD 2.9) days after admission (Table 1). The majority of these 214 patients were non-Hispanic White (*n* = 147, 69%) and male (*n* = 118, 55%), with a mean age of 58 years (SD 18.9). Most patients had suffered a stroke (*n* = 129, 60%), with 30% suffering TBI (*n* = 65), and 9% HIE (*n* = 20). Family members included spouses (*n* = 66, 31%), adult children (*n* = 75, 35%), parents (*n* = 31, 14%) or siblings and others (*n* = 42, 19%), and a majority of them were white (*n* = 142, 66%) and female (*n* = 138, 64%) with a mean age of 50.9 years (SD 16.1).

**Table 1:** Patient and Family characteristics.

	**Participants *n* = 214**
Patient age, mean (SD)	58.0 (18.9)
Patient gender, female, *n* (%)	96 (45%)
Patient race/ethnicity, non-white or hispanic, *n* (%)	67 (31%)
GCS at enrollment, mean (SD)	7.3 (2.6)
APACHE^*^at enrollment, mean (SD)	15.9 (4.4)
Disease category, *n* (%): - stroke	129 (60%)
- Traumatic brain injury (TBI)	65 (30%)
- Hypoxic-ischemic encephalopathy (HIE)	20 (9%)
Family age, mean (SD)	50.9 (16.1)
Family gender, female, *n* (%)	138 (64%)
Family race/ethnicity, non-white or hispanic, *n* (%)	62 (34%)
Family education <4yr college degree^**^, *n* (%)	116 (58%)
Family relationship—spouse/partner	66 (31%)
- Mother/father	31 (14%)
- Son/daughter	75 (35%)
- Sister/brother; other	42 (19%)

*Sample excludes subjects without a response to goals of care (n = 7)*.

* *APACHE (acute physiology and chronic health evaluation score): unknown for 34 patients (total), 21 all efforts, 6 comfort, 7 unsure*.

** *Family education: unknown for 15 patients (total), 7 all efforts, 5 comfort, 3 unsure*.

### Prioritized Goals-of-Care and Goal Concordance

**Figure 1:**
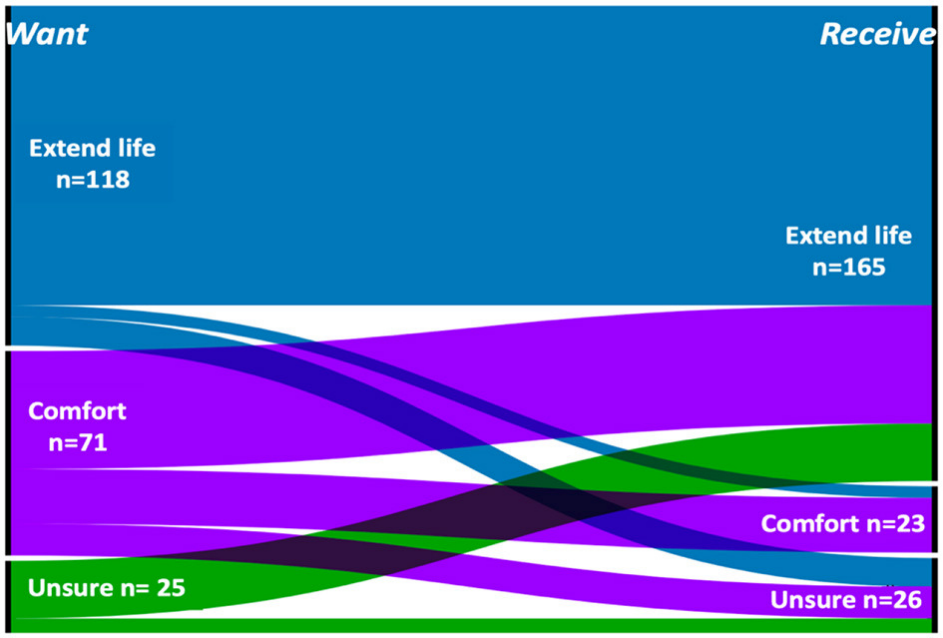
Alluvial diagram illustrating Goal concordance at enrollment. Families were asked what goals of care the patient would prioritize (“Want,” left boarder) and what type of care the patient was receiving at that time (“Receive,” right boarder); *n* = 214.

For these 214 patients with SABI, family members' assessment of patients' goals-of-care was extending life in 118 cases (55%); comfort in 71 (33%); and family members were unsure for 25 patients (12%). Goal concordance, meaning that the care the family assessed the patient was receiving was consistent with the care their family member assessed them as wanting, occurred in 104/118 (88%) of the patients presumed to want “extending life” and in 19/71 (27%) of those presumed to want “comfort.” Most of the families who were “unsure” of the patient's goals-of-care thought the patient was receiving “extending life” (20/25, 80%) while all others (5/25, 20%) were unsure of the focus of care the patient was receiving (Figure 1).

Overall, patients who were presumed to prioritize extending life compared to those presumed to prioritize comfort were younger (mean age 55.7 vs. 62.3 years), less likely to be non-Hispanic White (62 vs. 83%) and had lower clinical disease severity (mean APACHE score 14.9 vs. 17.6). Patients for whom family members reported they were unsure about patient priorities had a mean age of 56.3, 60% were non-Hispanic White, and mean APACHE was 15.4. After adjusting for potential confounders, race and clinical disease severity remained significantly associated with prioritized end-of-life values. Non-white patients had a 68% lower odds of prioritizing comfort vs. extending life (odds ratio, OR, 0.32, 95% confidence interval, CI, 0.14–0.73), and for every one-point increase in the APACHE score (=higher clinical severity), the odds of prioritizing comfort vs. endorsing extending life increased by 17% (OR 1.17, 95% CI 1.08–1.28; Table 2).

**Table 2:** Determinants of presumed prioritized healthcare goals at enrollment (*n* = 214).

**Covariate**	**Multivariate analysis;** ***n*** **=** **214**				
	***P* overall**	**Comfort** **(vs. extending life)**		**Unsure** **(vs. extending life)**	
		**OR**	**95% CI**	**OR**	**95% CI**
Age (per 10yr increase)	0.331	1.13	0.94–1.37	0.95	0.73–1.24
Non-white (vs. white)	**0.014**	**0.32**	**0.14**–**0.73**	0.45	0.13–1.51
Female (vs male)	0.407				
GCS (per 1pt increase)	0.826				
APACHE (per 1pt increase)	**0.001**	**1.17**	**1.08**–**1.28**	1.05	0.93–1.19

*Statistical significance by nominal regression. Multivariate model started with age/race, then added subsequent factors by forward selection (p < 0.05)*.

*P-values in gray indicate significance if the effect were added to the final multivariate model*.

*Bold indicates statistically significant (p < 0.05)*.

After adjusting for significant covariates via forward selection, the only significant association with goal concordance was having a family-assessed goal of extending life at enrollment (OR 23.62, 95% CI 10.19–54.77). We also found a trend suggesting a possible association with goal concordance in patients who were older after accounting for race and goals-of-care (see Table 3).

**Table 3:** Determinants of goal concordance at enrollment (*n* = 214).

**Covariate**	**Multivariate analysis;** ***n*** **=** **214**		
	***P***	**Concordance (vs. Discordance)**	
		**OR**	**95% CI**
Age (per 10yr increase)	0.066	1.23	0.99–1.54
Non-white (vs. white)	0.394	1.49	0.60–3.72
Female (vs. male)	0.838		
GCS (per 1pt increase)	0.084	0.88	
APACHE (per 1pt increase)	0.492		
Disease category	0.075		
TBI (vs. stroke)	0.056	0.39	
CA (vs. stroke)	0.087	0.28	
**Goal all efforts** **(vs. comfort)**	**0.003**	**23.62**	**10.19–54.77**

*Statistical significance by binary logistic regression*.

*Multivariate model started with age/race, then added subsequent factors by forward selection if p < 0.05*.

*P-values in gray indicate significance if the effect were added to the final multivariate model*.

*Bold indicates statistically significant (p < 0.05)*.

### Six-Month Outcomes

**Figure 2:**
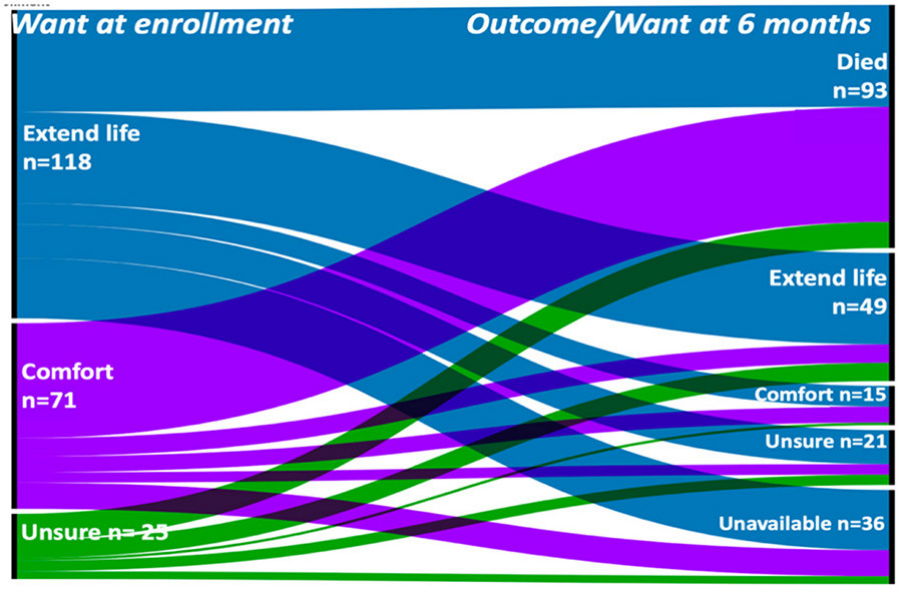
Alluvial diagram illustrating Change in prioritized goals over time. Families were asked what goals of care the patient would prioritize at enrollment (“Want at enrollment,” left boarder) and 6 months later (“Outcome/Want at 6 months,” right boarder). At 6 months, we had 85 survivors, 93 decedents and 36 non-respondents.

Of the 214 patients, 76 (36%) died in hospital and 17 more died over the ensuing 6 months. Six-month outcome was unavailable for 36 (26%), leaving 85 long-term survivors for whom family-assessed goals-of-care were available at a mean of 148 days (standard deviation, SD 43) after enrollment. These survivors had a mean age of 52 years (SD 18), a slight majority were non-Hispanic white (61%), male (55%) and had suffered a stroke (58%), TBI (36%) or HIE (6%). At this follow-up, family-assessed goals-of-care prioritized extending life for 58% of survivors (49/85), comfort for 18% (15/85), and family members were unsure of the patients' priorities for 25% (21/85). Figure 2 shows the distribution of goals at enrollment and at 6 months. Taking into account small numbers, multivariate regression suggested a significant association of age, ethnicity and disease category with prioritized healthcare goals at 6 months (Table 4). Compared to prioritizing life extension, the odds of prioritizing comfort was 1.6 times higher with every 10-year increase in age, and 4.8 times higher in patients with TBI compared to stroke. The odds of being unsure about goals (vs. prioritizing extending life) was three times higher for non-white patients compared to white patients.

**Table 4:** Determinants of presumed prioritized healthcare goals in the post-acute setting (148 days after enrollment).

**Covariate**	**Multivariate analysis; n** **=** **85**				
	***P* Overall**	**Comfort** **(vs. extending life)**		**Unsure** **(vs. extending life)**	
		**OR**	**95% CI**	**OR**	**95% CI**
Age (per 10yr increase)	**0.044**	**1.58**	**1.07**–**2.32**	1.05	0.73–1.52
Non-white (vs. white)	0.118	0.96	0.24–3.90	3.07	1.01–9.36
Female (vs. male)	0.845				
Disease	**0.033**				
TBI (vs. stroke)		**4.84**	**1.21–19.34**	0.52	0.12–2.27
CA (vs. stroke)		—	—	2.16	0.30–15.51

*Statistical significance by nominal regression*.

*Multivariate model started with age/race, then added subsequent factors by forward selection (p < 0.05)*.

*P-values in gray indicate significance if the effect were added to the final multivariate model*.

*Cell counts are too small to allow complete estimation for disease “CA vs. Stroke.”*

*Bold indicates statistically significant (p < 0.05)*.

## Discussion

In our cohort of 214 patients in the first week after severe acute brain injury (SABI), just over half of families felt that their loved one would prioritize extending life as much as possible. However, those family-assessed goals matched the family-reported care received for only 65% of patients. Life-sustaining treatment (LST) is often the default in hospital-level acute care unless a patient has specifically requested otherwise (13, 16). In the acute setting of SABI that is characterized by a high degree of prognostic uncertainty, national US guidelines even recommend “aggressive” therapy for those without advance directives to the contrary (17). Most families in our study report receiving care focused on extending life, regardless of goals, and goal concordance was accordingly more likely for patients who prioritized extending life. A presumed priority of comfort was more likely when patients were clinically sicker (by APACHE score), which may be related to their higher risk of mortality, although we do not know what information the family was given on the patient's chance of survival or recovery.

Our observation that non-Hispanic whites were more likely to prioritize comfort needs to be interpreted with caution as it was only significant for the comparison with “extending life” but not with “unsure.” We also did not collect detailed socio-economic characteristics, religious or cultural beliefs which might further confound this association. The observation is consistent with the literature that suggests higher prevalence of prioritizing aggressive care at the end-of-life in non-white compared to white patients and requires further research (18, 19).

One in four patients in our study may have been receiving unwanted aggressive care in the acute setting. It is possible that LST was provided with the mutual understanding of a time-limited trial, whereby family and clinicians have agreed on a period of aggressive interventions to see if the patient improves according to outcomes consistent with the patient's presumed goals-of-care (10, 20). In that case, this relatively high prevalence of aggressive care may be ethically justified as long as the prognostic uncertainty persists and as long as the time-limited trial is brought to a conclusion.

Future studies are needed to better understand the framework of goal concordance specifically in the setting of a time-limited trial after SABI. The possible associations of age, disease severity, and disease category also require further investigation. Goal concordance in older patients may be because they are more likely to have voiced their own goals prior to SABI, as described for the US population (21). Lower severity of SABI may account for more prognostic uncertainty leading to a trial of LST even in a patient who eventually might prioritize comfort. The trend toward a higher likelihood of goal concordance in patients with stroke could be related to the disease category itself, but also to the subspecialty of their medical providers (i.e., stroke neurologists vs. neurosurgeons or intensivists).

Six months after the acute event, a large proportion of family members were unsure of the patient's priorities. Given that SABI survivors are at high risk of re-hospitalization, these findings suggest important missed opportunities for improved communication between SABI survivors, their families and clinicians, even long after the event. Periodic re-evaluation of patient-centered goals and intentional conclusions to time-limited trials should be a routine part of post-SABI clinic visits (12, 22).

Our findings need to be considered in the setting of several important limitations. First, the single center design may limit generalizability of results. However, our center is the only comprehensive stroke and level 1 trauma center for a five-state region and is an academic county hospital serving wide variety of patients which may mitigate this limitation. Second, most of the patients were non-Hispanic white, and small numbers of non-white patients preclude analyses of separate minority races. Third, because patients were unable to communicate their own wishes or perspectives, we relied on families to provide substituted judgment. This does, however, reflect clinical practice where, if patients are unable to participate in decisions, goals-of-care are determined by family surrogates. Of note, only 22 (10%) of our patients had some type of pre-SABI advance directives documented in the EHR of which only half (*n* = 9) indicated any treatment preferences.

## Conclusion

The observed high prevalence of patients potentially receiving unwanted aggressive care after SABI may be justified in the acute setting as long as prognostic uncertainty exists and provided it is in the context of a well-implemented time-limited trial. The high prevalence of families who are unsure of their loved one's goals of care 6 months after SABI suggest missed opportunities in communication between clinicians, families and patients as well as missed opportunities for completion of time-limited trials in the post-acute setting. More research is needed to better understand goal concordance in both the acute and post-acute care setting in the context of a time-limited trial of life-sustaining treatment.

## Data Availability Statement

The raw data supporting the conclusions of this article will be made available by the authors, without undue reservation available to sincere requests.

## Ethics Statement

The protocol was approved by the ethical review board of the University of Washington (STUDY 00003393). The ethics committee waived the requirement of written informed consent for participation.

## Author Contributions

RR and CC: obtained funding and drafting of the manuscript. CC, RJ, and JC: supervison. JB: statistical analysis. RR, JB, JC, and CC: acquisition, analysis, or interpretation of data. RR, JC, RJ, and CC: concept and design. All authors critically revised the manuscript for important intellectual content.

## Funding

RR was supported by a grant of the Swiss National Science Foundation (P400PM_186732). CC received funding from a career development award from the National Institute of Neurological Disorders and Stroke (K23 NS099421). The researchers' funders had no role in the design, data analysis, interpretation or writing of the manuscript. All co-authors had full access to all the data in the study and had final responsibility for the decision to submit the publication.

## Conflict of Interest

The authors declare that the research was conducted in the absence of any commercial or financial relationships that could be construed as a potential conflict of interest. The reviewer, KD, declared a shared affiliation, though no collaboration, with three of the authors, RR, GB, and RJ to the handling editor.

## Publisher's Note

All claims expressed in this article are solely those of the authors and do not necessarily represent those of their affiliated organizations, or those of the publisher, the editors and the reviewers. Any product that may be evaluated in this article, or claim that may be made by its manufacturer, is not guaranteed or endorsed by the publisher.

## Abbreviations

The acronym SuPPOrTT stands for the 4 questions we asked clinicians and family members: - Do the patient or family require social, spiritual or emotional **Su**pport? - Does the patient have **P**ain or other distressing symptoms? - Does the family have concerns about **P**rognosis or treatment **O**ptions? - Do we need (**r**e-)address goals-of-care or **T**arget **T**reatment to patient-centered goals?
